# Single-molecule optical genome mapping of a human HapMap and a colorectal cancer cell line

**DOI:** 10.1186/s13742-015-0106-1

**Published:** 2015-12-29

**Authors:** Audrey S. M. Teo, Davide Verzotto, Fei Yao, Niranjan Nagarajan, Axel M. Hillmer

**Affiliations:** 1 Cancer Therapeutics and Stratified Oncology, Genome Institute of Singapore, 60 Biopolis Street, Singapore, 138672 Singapore; 2 Computational and Systems Biology, Genome Institute of Singapore, 60 Biopolis Street, Singapore, 138672 Singapore

**Keywords:** Optical mapping, Genomic mapping, Cancer genome, Genome structure, Single-molecule restriction mapping

## Abstract

**Background:**

Next-generation sequencing (NGS) technologies have changed our understanding of the variability of the human genome. However, the identification of genome structural variations based on NGS approaches with read lengths of 35–300 bases remains a challenge. Single-molecule optical mapping technologies allow the analysis of DNA molecules of up to 2 Mb and as such are suitable for the identification of large-scale genome structural variations, and for *de novo* genome assemblies when combined with short-read NGS data. Here we present optical mapping data for two human genomes: the HapMap cell line GM12878 and the colorectal cancer cell line HCT116.

**Findings:**

High molecular weight DNA was obtained by embedding GM12878 and HCT116 cells, respectively, in agarose plugs, followed by DNA extraction under mild conditions. Genomic DNA was digested with *Kpn*I and 310,000 and 296,000 DNA molecules (≥150 kb and 10 restriction fragments), respectively, were analyzed per cell line using the Argus optical mapping system. Maps were aligned to the human reference by OPTIMA, a new glocal alignment method. Genome coverage of 6.8× and 5.7× was obtained, respectively; 2.9× and 1.7× more than the coverage obtained with previously available software.

**Conclusions:**

Optical mapping allows the resolution of large-scale structural variations of the genome, and the scaffold extension of NGS-based *de novo* assemblies. OPTIMA is an efficient new alignment method; our optical mapping data provide a resource for genome structure analyses of the human HapMap reference cell line GM12878, and the colorectal cancer cell line HCT116.

## Data description

The analysis of human genome next-generation sequencing (NGS) data largely focuses on the detection of single nucleotide variants (SNVs), and insertions and deletions of a few base pairs (indels). Larger genome structural variations (SVs) that can result in copy number variations (CNVs) affect up to 13 % of the human genome [1]. However, the detection of SVs, in particular of copy number neutral events such as inversions, 'cut and paste' insertions, or balanced translocations through NGS analysis is less straightforward [2]. A particular problem lies in the short read length of 35–300 bases of the most commonly used NGS approaches, which does not, in many cases, allow unambiguous mapping of the respective reads to the human reference genome. This is relevant since transposable elements with their sequence similarities account for a large proportion of SVs in the human genome [3], and rearrangement points tend to occur in repetitive sequences [4]. In contrast, single-molecule optical mapping technologies label large DNA fragments of up to 2 Mb that allow the identification of large SVs and *de novo* assembly of genomes [5–9]. The length of single DNA molecules provides a higher sensitivity for the identification of large SVs with rearrangement points within repetitive sequences compared to standard NGS approaches.

Optical mapping is a light microscope-based technique for constructing ordered physical maps of restriction enzyme recognition sites across a genome. It has been applied to characterize the structure of the human genome [8–10] but only a small fraction of the raw optical maps is usually used for mapping. We aimed to improve the efficacy of data analysis to allow greater scalability of this approach. Here we present optical mapping data for two human genomes: the HapMap cell line GM12878, and the colorectal cancer cell line HCT116.

High molecular weight (HMW) DNA was extracted from the human cell lines GM12878 and HCT116 as follows. Cells were embedded in agarose plugs at a concentration of approximately 10^7^ cells/ml by mixing a cell suspension in phosphate buffered saline (PBS) with a 1 % low melting point agarose–PBS solution, dispensing the mixture into plug molds (Bio-Rad Laboratories, Inc.) and allowing the plugs to solidify completely. Cell lysis within the agarose plugs was performed by immersing the plugs in 5 ml of lysis buffer (0.5 M EDTA, pH 9.5; 1 % lauroyl sarcosine, sodium salt; proteinase K, 2 mg/ml) at 50 °C for 2 days, with gentle agitation and a change of lysis buffer in between. The plugs were then washed three times with 45 ml of 1X TE buffer (pH 8.0) per wash with gentle rocking. The DNA that remained immobilized within the agarose plugs was released by melting the agarose at 70 °C for 7 min, followed by incubation with β-agarase in 1X TE buffer (pH 8.0) at 42 °C overnight. Argus 10X Loading Buffer (OpGen Inc) was added to the sample (to approximately 1X concentration), and incubated overnight at room temperature. The HMW DNA was further diluted in Argus Dilution Buffer (OpGen Inc) and incubated overnight at 37 °C before determining the DNA length and concentration on Argus QCards (OpGen Inc).

**Fig. 1:**
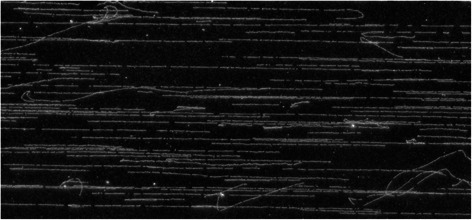
Representative optical map of GM12878. DNA molecules were stretched and immobilized onto a glass MapCard surface with the aid of a channel-forming device, cut by *Kpn*I, stained, and visualized by fluorescence imaging. Interrupted linear stretches indicate DNA digested by *Kpn*I. Whirly, non-linear, short, and disjointed DNA molecules are filtered out by the image processing software

Argus MapCards were assembled following the manufacturer’s protocol, using Argus consumables and reagents (OpGen Inc). HMW DNA prepared as described above was allowed to flow through a high density channel-forming device (CFD), which was placed on an Argus MapCard surface attached to an Argus MapCard II. This resulted in single DNA molecules being stretched and immobilized on the surface. The CFD was removed, a cap was placed over the DNA, and reagents (antifade, buffer, enzyme, stain) were loaded into the MapCard reservoirs. The assembled MapCard was placed in the Argus MapCard Processor where digestion with *Kpn*I enzyme (Table 1) and staining of DNA molecules occurred in an automated process. The MapCard was removed from the Argus Mapcard Processor and sealed, then placed in the Argus Optical Mapper and set up for automatic data collection as described previously [5]. Argus Mapper was used to image DNA molecules and corresponding restriction fragments by fluorescence microscopy (Fig. 1). The Argus System merged images into channel images and labeled DNA molecules of 150 kb to 2 Mb. Restriction enzyme cut sites were detected as gaps in linear DNA molecules, and the size of each restriction fragment between adjacent cut sites was determined. The Mapper filtered out non-linear distorted fragments and small molecules, identified gaps between fragments, and measured the size of retained high quality fragments. Data from DNA molecules with at least 10 fragments and quality scores of 0.2 were collected from 4 and 6 MapCards for GM12878 and HCT116 cell lines, respectively.

**Table 1:** *In silico* analysis of restriction enzyme cutting statistics for the human reference genome (hg19)

Enzyme	Usable DNA fragments (%)			Average fragment size (kb)	Maximum fragment size (kb)	#Fragments >100 kb
	5–20 kb	6–15 kb	6–12 kb			
*Afl*II	13.3	5.48	5.43	4.47	143.96	4
*BamH*I	99.22	92.95	92.9	7.92	153.92	21
***Kpn*** **I**	**99.95**	**99.88**	**99.51**	**9.98**	**171.76**	**65**
*Nco*I	0.08	0.03	0.03	3.81	164.18	2
*Nhe*I	99.86	98.97	90.75	10.23	204.75	88
*Spe*I	99.28	96.71	94.55	7.27	311.48	101
*Bgl*II	2.33	0.81	0.8	3.71	109.69	1
*EcoR*I	2.21	0.79	0.79	3.67	86.14	0
*Mlu*I	0.34	0.01	0.01	135.32	2276.59	8295
NdeI	5.9	1.78	1.78	3.19	105.86	1
*Pvu*II	0.03	0.02	0.02	2.66	173.76	6
*Xba*I	2.75	1.15	1.15	3.58	146.27	2
*Xho*I	17.02	6.37	2.21	23.78	430.88	3269

To select the restriction enzyme that cuts the human genome to maximize the fraction of fragments resulting in informative maps, the human genome was cut *in silico* with 13 commonly used restriction enzymes based on their canonical cutting sites. Usable restriction fragment sizes were defined as 5–20 kb, 6–15 kb, and 6–12 kb, since smaller DNA fragments do not allow accurate size estimates, and longer fragments can result in maps with too few fragments. *Kpn*I was selected based on its high fraction of usable DNA fragments (highlighted in bold)

We obtained 309,879 and 296,217 maps (fragmented DNA molecules) for GM12878 and HCT116, respectively; these had ≥10 fragments and were ≥150 kb in length (Tables 2 and 3), and were used as inputs for alignment by OPTIMA [11–13]. These criteria are more inclusive compared to the default parameters for alignment by the state-of-the-art algorithm Gentig v.2 (OpGen Inc) [5, 14]. MapCard output for maps with these criteria ranged between 3,744 and 93,896 maps. Average fragment sizes were 16.4 kb for GM12878, and 15.7 kb for HCT116. OPTIMA allowed alignment of 20.9 and 18.1 % of maps with these criteria, significantly more than by using Gentig [12]. Average digestion rates were estimated to be 0.66 and 0.691 (cuts), and extra-cutting rates were estimated to be 0.751 and 0.774 cuts per 100 kb for GM12878 and HCT116, respectively.

**Table 2:** Summary of MapCard statistics of GM12878

MapCard ID	F^a^	Input maps^b^ (theoretical genome coverage)	Average Argus quality score	Average DNA molecule size (kb)	Average # of fragments	Average fragment size (kb)	OPTIMA alignment rate	Yield (genome coverage)^c^	Average digestion rate^c^	Average false/ extra cut rate^c^	Ratio small missing fragments (≤2 kb)^c^
21157LB	(r)	73365 (7.2×)	0.50	295	18	16.5	0.253	2.0×	0.659	0.736	0.139
	(s)	38483 (4.7×)	0.53	368	22	17.0	0.357	1.7×	0.650	0.733	0.133
21159LB	(r)	75761 (7.6×)	0.47	300	17	17.4	0.190	1.6×	0.628	0.723	0.129
	(s)	41236 (5.1×)	0.50	370	21	17.8	0.268	1.3×	0.618	0.718	0.124
21431LB	(r)	93896 (8.6×)	0.52	274	17	15.8	0.200	1.9×	0.676	0.773	0.187
	(s)	43667 (5.1×)	0.54	348	21	16.3	0.303	1.5×	0.665	0.768	0.184
21443LB	(r)	66857 (6×)	0.51	271	17	15.8	0.192	1.3×	0.674	0.771	0.175
	(s)	29991 (3.5×)	0.53	346	21	16.3	0.292	1.0×	0.661	0.772	0.168
Total	(r)	309879 (29.4×)	0.50	285	17	16.4	0.209	6.8×	0.660	0.751	0.158
	(s)	153377 (18.3×)	0.52	359	21	16.9	0.310	5.5×	0.649	0.747	0.152

^a^r: inclusion of DNA molecules with ≥10 fragments and ≥150 kb in length; s: inclusion of DNA molecules with ≥12 fragments and ≥250 kb in length

^b^fragmented DNA molecules

^c^of OPTIMA aligned data

**Table 3:** Summary of MapCard statistics of HCT116

MapCard ID	F^a^	Input maps^b^ (theoretical genome coverage)	Average Argus quality score	Average DNA molecule size (kb)	Average # of fragments	Average fragment size (kb)	OPTIMA alignment rate	Yield (genome coverage)^c^	Average digestion rate^c^	Average false/ extra cut rate^c^	Ratio small missing fragments (≤2 kb)^c^
17182LA	(r)	10911 (0.9×)	0.33	257	16	15.7	0.040	0.04×	0.661	1.288	0.170
	(s)	3744 (0.4×)	0.33	351	20	17.7	0.040	0.02×	0.628	1.226	0.190
17184LA-2	(r)	55719 (5.7×)	0.43	305	19	16.3	0.180	1.1×	0.678	0.760	0.197
	(s)	28658 (3.7×)	0.45	390	23	17.2	0.250	0.9×	0.669	0.737	0.199
17185LA	(r)	56879 (5.4×)	0.55	285	19	14.7	0.240	1.5×	0.705	0.756	0.219
	(s)	28003 (3.4×)	0.59	365	24	15.1	0.352	1.2×	0.696	0.739	0.217
17186LA-3	(r)	52984 (5.8×)	0.54	328	20	16.0	0.327	2.0×	0.696	0.677	0.167
	(s)	31588 (4.3×)	0.56	404	25	16.4	0.423	1.7×	0.688	0.671	0.163
17187LA	(r)	88730 (7.8×)	0.45	264	18	14.8	0.115	1.0×	0.692	0.940	0.195
	(s)	36018 (4.2×)	0.46	349	22	15.8	0.171	0.7×	0.678	0.919	0.188
14593LB	(r)	30994 (2.7×)	0.39	261	14	18.9	0.059	0.2×	0.626	0.847	0.161
	(s)	10944 (1.2×)	0.39	337	17	20.2	0.086	0.1×	0.597	0.869	0.151
Total	(r)	296217 (28.3×)	0.47	287	18	15.7	0.181	5.7×	0.691	0.774	0.191
	(s)	138955 (17.2×)	0.50	372	23	16.5	0.271	4.6×	0.682	0.749	0.188

^a^r: inclusion of DNA molecules with ≥10 fragments and ≥150 kb in length; s: inclusion of DNA molecules with ≥12 fragments and ≥250 kb in length

^b^fragmented DNA molecules

^c^of OPTIMA aligned data

Although enzyme selection, data filtering protocols and alignment methods greatly influence data metrics, we compared our data with an optical mapping study of two human cancer genomes (Ray and colleagues; [8]). The average DNA molecule size of our GM12878 and HCT116 maps with ≥12 fragments and ≥250 kb in length were 359 and 372 kb, respectively. The Ray et al. data had average DNA molecule sizes of 434 and 421 kb, respectively. The aligned coverage of the human genome for GM12878 and HCT116 was 5.5× and 4.6×, respectively, while the Ray et al. data gave 37× and 25× coverage. Estimated digestion rates were 65 and 68 % with *Kpn*I for GM12878 and HCT116, respectively, while digestion rates were 83 and 82 % with *Swa*I for the Ray et al. data. For GM12878 and HCT116 we estimated 0.747 and 0.749 extra cuts per 100 kb, respectively, while the data of Ray et al. showed 0.168 and 0.233 extra cuts per 100 kb.

While GM12878 has been analyzed by paired-end sequencing [15], resolving the genome structure is restricted by the limitations of short-read sequencing. The data presented here is a resource to define the genome structure of this HapMap cell line, as well as that of HCT116, a commonly used colorectal cancer cell line. Cancer genomes are known to be rearranged to various extents. The interpretation of epigenetic alterations and mutations in non-coding but regulatory regions of the genome will only be accurate if they are seen in the correct genomic context, i.e. in the sample-specific genome structure. This requires methodologies like single-molecule optical mapping to resolve the genome structure beyond what is possible with short-read NGS data.

## Availability and requirements of software used

OPTIMA can be downloaded from GigaScience DB [13] at http://dx.doi.org/10.5524/100165 and at http://www.davideverzotto.it/research/OPTIMA. The software requirements are Oracle Java SE Development Kit 7+, Apache Commons Math 3.2 JAR library, and CERN Colt 1.2.0 JAR library.

## Availability of supporting data and materials

The datasets supporting the results of this Data Note are available in the GigaScience repository, GigaDB [16]. Also, the supporting material for the OPTIMA tool used for alignment of data in this paper can be found in GigaDB [13].

## Abbreviations

CFD: channel-forming device
CNV: copy number variation
HMW: high molecular weight
indel: insertion or deletion of a few base pairs
NGS: next-generation sequencing
PBS: phosphate buffered saline
SNV: single-nucleotide variant
SV: structural variation
